# Association of Pre-Eclampsia with Carotid Artery Intima–Media Thickness and Non-Alcoholic Fatty Liver Disease

**DOI:** 10.3390/jcm7090275

**Published:** 2018-09-12

**Authors:** Behzad Memari, Niloofar Moghiseh, Farnaz Mohammadian, Mahsa Ghajarzadeh, Hadi Ghoreishian

**Affiliations:** 1 Department of Radiology, Zanjan University of Medical Science, Zanjan 1467933811, Iran; bdmemari@zums.ac.ir (B.M.); Niloofarmoghiseh@gmail.com (N.M.); 2 Department of Obstetrics and Gynecology, Zanjan University of Medical Science, Zanjan 1467933811, Iran; farnazmohamadian@zums.ac.ir; 3 Department of Universal Council of Epidemiology (UCE), Universal Scientific Education and Research Network (USERN), Tehran 1467933811, Iran; m.ghajarzadeh@gmail.com

**Keywords:** thickness of the intima–media carotid artery, non-alcoholic fatty liver disease, pre-eclampsia, pregnant women

## Abstract

Atherosclerosis and non-alcoholic fatty liver disease (NAFLD) are considered important complications of pre-eclampsia. This study was conducted to determine the association of pre-eclampsia with non-alcoholic fatty liver disease and the association of pre-eclampsia with bilateral intima–media thickness (IMT; right and left), separately. Twenty-one pregnant women with pre-eclampsia and 21 normal pregnant women were enrolled in the present study. The right and left intima–media thicknesses of carotid arteries were evaluated using Doppler sonography. The diagnosis of NAFLD was based on sonography. Linear and binary logistic regression analyses were performed to evaluate the association between pre-eclampsia and related outcomes. The mean right IMT was determined as 0.60 ± 0.07 mm in women with pre-eclampsia and 0.51 ± 0.08 mm in normal pregnant women (*p* = 0.001). On the other hand, the mean left IMT was 0.59 ± 0.09 mm in women with pre-eclampsia and 0.50 ± 0.10 mm in normal pregnant women (*p* = 0.003). The frequencies of NAFLD in women with pre-eclampsia and normal pregnant women were found to be 66.7% and 23.8% respectively (*p* = 0.006). Multivariate linear regression analysis revealed an association between pre-eclampsia and right (*p* = 0.014) and left (*p* = 0.019) IMT, without removing the effects of other confounding variables. Binary regression analysis (multivariate) did not confirm an independent association between pre-eclampsia and NAFLD. Pre-eclampsia exhibited a direct and independent association with right and left IMT. Although the prevalence of NAFLD was significantly higher in women with pre-eclampsia, pre-eclampsia was not an independent predictor for NAFLD.

## 1. Introduction

Nonalcoholic fatty liver disease (NAFLD) is a progressive condition, and is characterized by excessive fat accumulation in the form of triglycerides (steatosis) in the liver. It is noteworthy that metabolic disorders such as obesity, diabetes mellitus, and hyperlipidemia are revealed to be predictive factors for occurrence of NAFLD [1,2,3]. Pre-eclampsia is a syndrome of pregnancy that affects almost all organs of the body, and these changes ultimately lead to multiple organ involvement and the emergence of a range of clinical symptoms. These clinical manifestations may be difficult to identify, and even pathophysiologic disturbances may be risky for the mother and the fetus [4,5]. Pre-eclampsia is described as raised blood pressure after 20 weeks of gestation in patients with previously normal blood pressure. On the other hand, patients who suffer from proteinuria value ≥0.3 g in a 24-h urine sample, with a protein (mg/dL)/creatinine (mg/dL) ratio ≥ 0.3 or a urine dipstick protein of 1+ are more susceptible to pre-eclampsia [6,7,8,9,10]. Moreover, it should be taken into consideration that diagnostic criteria for pre-eclampsia in woman with new-onset hypertension without proteinuria have been defined in the following way: Platelet count < 100,000/μL, serum creatinine level > 1.1 mg/dL or a doubling of serum creatinine (if evidence of other kidney disease is not available), an increased level of liver transaminases to least twice the normal level, and pulmonary edema, as well as cerebral or visual symptoms [2,11,12]. In addition, severe hypertension or proteinuria is associated with a definite diagnosis of pre-eclampsia and its adverse outcomes. Potential risk factors of pre-eclampsia include age, number of pregnancies, race, ethnicity, genetic background, obesity, and multiple pregnancies, as well as smoking [13,14].

Atherosclerosis is a significant pathologic cause of cardiovascular (CV) and cerebrovascular diseases that have remarkable impacts on morbidity and mortality. Therefore, the primary prevention of these diseases has become the focus of current studies. In parallel, studies have revealed that carotid ultrasonography is more sensitive than the coronary artery calcification score (CACS) for the diagnosis of subclinical atherosclerosis. Accordingly, carotid intima–media thickness (IMT) ultrasonography may play a key role in detection of subclinical atherosclerosis as an accessible and reliable method [15,16]. Ultrasound evaluations of fatty changes in non-alcoholic fatty liver and liver elasticity have been suggested as valid tools for assessing the pathologic status of patients with liver diseases. Sonographic changes in fatty liver disease include increased liver echogenicity and increased ultrasound absorption, which leads to the unclear image in the posterior part of the liver, liver veins, and the portal vein [16,17]. A study in the Netherlands suggested that pre-eclampsia is associated with an increased risk of cardiovascular events in the long term, and in women with pre-eclampsia, carotid IMT and femoral arteries are at increased risk of presenting early atherosclerosis as compared to normal women. Another study found that a brief increase in blood pressure, triglycerides, and homocysteine were associated with pre-eclampsia among women; in addition, an increase in the thickness of the carotid intima–media elevated the risk of pre-eclampsia [18]. Another study revealed that early pre-eclampsia could be associated with premature cardiovascular disease, with increased femoral artery IMT and elevated cardiovascular disease markers one year after pre-eclampsia. However, as another study indicated, the intima–media thickness did not increase in 17 women with pre-eclampsia, indicating a transient vascular response in these patients. Only hypertension, increased body mass index (BMI), and elevated serum triglycerides and inflammatory markers were observed in women suffering from pre-eclampsia, as compared to a control group [19].

We carried out a cross-sectional survey to determine the association of pre-eclampsia with carotid IMT and NAFLD.

## 2. Experimental Section

### 2.1. Ethics Approval

This study was approved by the Hospital Ethical Committee of Zanjan University of Medical Science, and all experiments were performed in accordance with the principles of the Declaration of Helsinki. Written informed consent was obtained from all patients (ZUMS.REC.2016.05).

### 2.2. Study Population

The study population included healthy pregnant women and pregnant women with proven pre-eclampsia in the third trimester of pregnancy. A total sample size (including both healthy pregnant women and pregnant women with pre-eclampsia) of 42 subjects was calculated according to the following formula:

$$n=\frac{{\left(Z_{1\;-\;\alpha /2}+Z_{1\;-\;\beta }\right)}^{2}\times \left(S_{1}^{2}+\;S_{2}^{2}\right)}{{\left(\mu_{1}-\;\mu_{2}\right)}^{2}}\;\alpha =0.05\;\beta =0.1\;\mu_{1}=0.63\;\mu_{2}=0.52\;S_{1}=0.14\;S_{2}=0.06\;n=20.086=21\;n=\frac{{\left(1.96+084\right)}^{2}\times \left(S_{1}^{2}+S_{2}^{2}\right)}{{\left(\mu_{1}-\mu_{2}\right)}^{2}}\;$$

where *n* = sample size; *z* = standard normal deviation; and *Z*_1−*α*/2_ = 1.96 for a significance level of 95%. *α* = 0.05 and *Z*_1−*β*_ = 0.84 when *β* = 0.10 (study power = 80%). (*μ*_1_ − *μ*_2_) = effect size; *S*_1_ = standard deviation in the first group; and *S*_2_ = standard deviation in the second group.

The inclusion criteria included the absence of pre-eclampsia for healthy pregnant women, the presence of pre-eclampsia for pregnant women in the pre-eclampsia group, and pregnancy in the third trimester for both groups. Moreover, exclusion criteria included risk factors for atherosclerotic diseases such as diabetes, hyperlipidemia, and smoking and alcohol consumption in both groups. In the current study, 21 pregnant women in the third trimester with normal pregnancy and 21 pregnant women with pre-eclampsia in the third trimester were enrolled in the study. After obtaining written consent, the questionnaire was adjusted according to personal characteristics and risk factors including name, age, spouse age, gestational age on the basis of the last menstruation and ultrasound, number of previous pregnancies, number of childbirth, weight, height, history of smoking, alcohol consumption, history of diabetes, history of hypertension, and history of gestational diabetes, as well as history of pre-eclampsia and current blood pressure. In addition, IMT was measured by color Doppler sonography and the liver was also evaluated by an ultrasound radiologist for the presence of NAFLD. Furthermore, hemoglobin, platelet, lactate dehydrogenase (LDH), aspartate transaminase (AST), alanine aminotransferase (ALT), low-density lipoprotein (LDL), high-density lipoprotein (HDL), triglyceride (TG), cholesterol (CHOL), creatinine, and protein values, as well as 24-h urine volumes, were recorded in the two groups using biochemical tests.

A carotid ultrasound is performed on all patients by a high-resolution B-mode ultrasound system via linear-array transducer. The carotid intima–media thickness is defined as the distance between the lumen–intima and the media–adventitia of the carotid arterial wall on the ultrasound in a longitudinal view. Measurements were conducted from the far wall of the common carotid artery at the level of the proximal part of the bulbus arteriosus with 1–2 cm distance from bifurcation within a region free of plaque. The average value of right and left carotid arteries was considered.

Fatty liver was diagnosed with trans-abdominal ultrasonography using a 5-MHz probe as a surrogate to liver biopsy (gold standard method for hepatic steatosis). The ultrasound findings in cases of fatty liver include increased hepatorenal echo contrast, liver brightness, vascular blurring, and deep attenuation. Trans-abdominal ultrasonography was implemented by a consultant radiologist who was blinded to the subjects’ clinical details or laboratory findings.

### 2.3. Statistical Analysis

The data were analyzed by SPSS software (Version 20 SPSS Inc., Chicago, IL, USA) after assigning appropriate codes. In descriptive statistics, the mean and standard deviations were used for quantitative and continuous variables. Furthermore, qualitative variables were reported by percentage and frequency in the tables. Mean analysis was presented in two groups by independent *t* test. A chi-squared test was applied to determine the relationship between independent nominal variables and the outcome. Moreover, in order to investigate the relationship between pre-eclampsia and IMT, linear regression was employed apart from the effects of other variables, including age variables, body mass index, blood cholesterol levels, and serum AST and ALT levels that were included in the study. For this model, the beta coefficient was standardized and also t-statistics and significant levels for all variables were reported. Additionally, in order to investigate the relationship between the twofold outcome of pre-eclampsia and NAFLD, a logistic regression was applied in a multivariate model that included age, body mass index, blood cholesterol levels, and AST and ALT levels. By applying this model, odds ratios, Wald statistics and significance levels were provided for interpretation of data. On the other hand, the receiver operating characteristic (ROC) curves were plotted to determine the differences of right and left IMT for pre-eclampsia. A *p*-value < 0.05 was considered to be statistically significant, and the area under the curve (AUC) was plotted for determining the differences between left and right IMT.

## 3. Results

The basic characteristics of the participants in the study were examined based upon the type of variables. The quantitative and continuous variables (Table 1) were given in terms of mean and standard deviation, while the qualitative and ranking variables (Table 2) were calculated as percentages. In this study, the mean age of mothers was recorded as 31.43. Other mean values collected were gestational age (33.67 weeks), body mass index (31.47 kg/m^2^), right carotid artery intima–media thickness (0.55 mm), left carotid artery IMT (0.54 mm), non-alcoholic fatty liver (54.8%), history of hypertension (90.5%) and history of pre-eclampsia (83.3%). Other details are summarized in Table 1 and Table 2.

Means were compared in two groups by independent *t* test. Variables such as age, body mass index, systolic and diastolic blood pressure, blood cholesterol levels, serum ALT and AST levels, urine protein volume, 24-h urine, right carotid artery intima–media thickness, and left carotid artery intima–media thickness showed a significance level below 0.2 in both groups. Other details are summarized in Table 3.

The chi-squared test was used to determine the relationship between nominal and outcome variables. Based on data presented herein, prior histories of hypertension, pre-eclampsia, and NAFLD were found to be significantly different (below 0.2) in the comparison of both groups. Other details are shown in Table 4.

In order to investigate the association between pre-eclampsia and left and right carotid artery IMT apart from the effects of other variables, linear regression was employed by which a number of variables including the right carotid artery intima–media thickness, the left carotid artery IMT as outcome variables, age, BMI, and serum cholesterol level, as well as serum AST and ALT level, exhibited a significance level below 0.2 in univariate models. The findings suggested that pre-eclampsia was significantly correlated with the carotid artery IMT (left and right), even if the effects of other variables are exactly eliminated. Pregnant women with pre-eclampsia had higher carotid artery IMT as compared to healthy pregnant women (Table 5 and Table 6).

In order to evaluate the correlation of pre-eclampsia with NAFLD apart from the effects of other variables, a multivariate logistic regression model was performed in which age, body mass index, blood cholesterol levels and AST and ALT levels were entered. Unlike the single-variable model, there was no significant association between healthy pregnant women and pregnant women with pre-eclampsia and NAFLD by eliminating the effects of other variables. The results are presented in Table 7.

**Figure 1:**
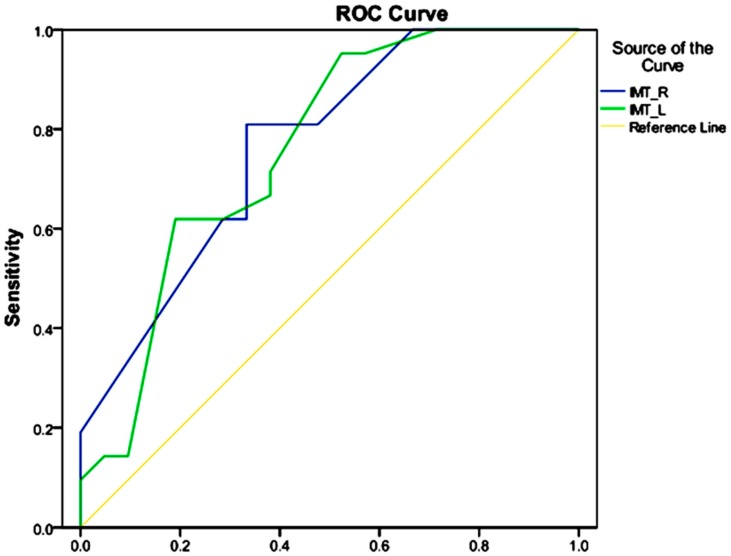
ROC curves for determining power of the IMT in the right and left carotid arteries in the diagnosis of pre-eclampsia.

To determine the separation power of the carotid artery IMT for diagnosis of pre-eclampsia, ROC curves were plotted (Figure 1). All significant levels were considered as 0.05 for determining the difference between variables. The areas under the ROC curves for the right and left carotid artery IMT were determined to be 0.766 and 0.759, respectively, where considered acceptable in the diagnosis of pre-eclampsia.

## 4. Discussion

The findings of our study depicted that IMT of the right and left carotid arteries in pregnant women with pre-eclampsia was significantly higher than healthy pregnant women. These results remained significant when the effects of age variables, body mass index, blood cholesterol and serum ALT and AST levels were eliminated. In other words, pre-eclampsia is independently and directly associated with an increase in IMT of the right and left carotid arteries. Increased carotid artery IMT has been previously revealed as an important predictor of atherosclerosis, which can increase the risk of cardiovascular disease and cerebrovascular disease. This clinically emphasizes the early diagnosis and treatment of pre-eclampsia. The treatment of pre-eclampsia due to the risk of eclampsia is an undeniable necessity in pregnant women; however, clinical concerns regarding pre-eclampsia cannot be limited to acute and immediate effects. Studies have revealed that the determination of carotid artery IMT could aid in identifying individuals with subclinical or advanced atherosclerosis, and even assist in minimizing the severity of this disorder in a non-invasive manner. Yuan et al., in a meta-analysis, suggested carotid IMT as a predictor of vascular events in the future and, in line with our study results, concluded that carotid artery IMT significantly increased in pregnant women with pre-eclampsia as compared to healthy pregnant women. It is clear that carotid artery IMT, even in healthy people, can be influenced by increased blood pressure; nevertheless, studies have demonstrated that this event can be linked to the effects of systemic and pulmonary circulation in pre-eclampsia. The importance of increasing the thickness of carotid artery intima–media is so high that some researchers recommend measuring this indicator in pregnant women [20].

On the other hand, American cardiac studies also report that the incidence of cerebrovascular disease (CVA) in women is higher than males, in contrast to the high incidence of heart disease in men. In this context, 60% of deaths from cerebrovascular disease in the United States occur in women. Although women have many common risk factors with men for the development of cerebrovascular disease, they have increased risk of cerebrovascular disease due to factors such as the use of the contraceptive pill and complications of pregnancy. This indicates that the carotid artery IMT play a key role in identifying vulnerable women for the development of cerebrovascular events [21]. We also examined the diagnostic value of the right and left carotid artery IMT in patients with pre-eclampsia. The ROC curves provided an area under the curve of 0.766 for the right carotid artery IMT, while AUC value was calculated to be 0.759 for the left carotid artery IMT, values considered acceptable in the diagnosis of pre-eclampsia. Akhter et al. obtained an AUC value of 0.25% for carotid artery IMT, which suggests that model did not perform appropriately [22]. However, this method is most likely used to predict a future event and cannot play a decisive role in our study. Because, we studied the effect of pre-eclampsia on the carotid artery IMT with pre-eclampsia, the independent effects of other variables could potentially contribute to increasing carotid artery IMT of both sides. A study by Hammoud et al. demonstrated that the prevalence of NAFLD was significantly higher in pregnant women with pre-eclampsia compared with healthy pregnant women [23]; as the aforementioned study indicated, the prevalence of NAFLD among pregnant women with pre-eclampsia was approximately three times that of NAFLD in healthy pregnant women. However, the relationship between NAFLD and pre-eclampsia was not statistically significant; when the effects of variables such as age variables, body mass index, blood cholesterol level, and AST and ALT levels are eliminated from investigation, the relationship still can be affected by other variables. The current study highlighted the effect of pre-eclampsia on fatty liver; nonetheless, this effect was applied through other variables and independent effects of pre-eclampsia was not available. It is expected that age increases the prevalence of non-alcoholic fatty liver and is also linked to the incidence of pre-eclampsia. Therefore, age as a confounding variable could justify a higher incidence of NAFLD in subjects with pre-eclampsia. Moreover, BMI can play a key role as an intermediate variable in the NAFLD–pre-eclampsia relationship. Obesity is one of the most important risk factors of NAFLD and pre-eclampsia; pre-eclampsia has a significant correlation with obesity.

Additionally, it is not clear in the literature whether pre-eclampsia leads to an increase in IMT and atherosclerotic changes or not. These are only novel criteria which could help clinicians for early diagnosis of the pre-eclampsia. Nevertheless, the diagnosis of disease may be sometimes more complicated in pregnant women than is the case in textbook presentations, as symptoms can overlap with those of other complications of pregnancy.

## 5. Conclusions

In summary, the IMT of the right and left carotid arteries in pregnant women with pre-eclampsia was found to be significantly higher when compared with healthy pregnant women, and pre-eclampsia was directly and independently linked to IMT of the right and left carotid arteries. Hence, carotid artery IMT can lead to an increase in atherosclerosis, risk of cardiovascular disease, and cerebrovascular events. This event clinically emphasizes the early diagnosis and treatment of pre-eclampsia. Furthermore, the prevalence of NAFLD in pregnant women with pre-eclampsia was markedly higher than in healthy pregnant women. However, this association can be affected by other variables, while independent effects of pre-eclampsia were not obtained in the present study. Overall, pre-eclampsia was not found to be an independent predictor for NAFLD.

In this study, one limitation was the number of pre-eclampsia patients included. We also did not investigate the relationship between the severity of pre-eclampsia and carotid artery IMT and NAFLD. Moreover, pregnant women with pre-eclampsia were not followed up after childbirth and thus we did not investigate the long-term effects of pre-eclampsia on the IMT of the right and left carotid arteries and NAFLD. Another limitation was the cross-sectional nature of the study. This study did not seek to discover the association between causes of disease and their effects; in order to discover these relationships, prospective cohort studies are needed.

## Figures and Tables

**Table 1:** Basic characteristics of the studied population in terms of mean and standard deviation.

Variable	Mean ± SD (*n* = 42)
Mother’s age (years)	31.43 ± 5.58
Gestational age (week)	33.67 ± 2.88
BMI, kg/m^3^	31.47 ± 3.63
Systolic blood pressure (mmHg)	114.28 ± 16.03
Diastolic blood pressure (mmHg)	74.28 ± 14.51
Hb, g/dL	12.3 ± 1.7
PLT × 10^3^/mL	192.5 ± 54.4
TG, mg/dL	319.8 ± 108.6
CHOL, mg/dL	217.5 ± 58.5
LDL, mg/dL	127.8 ± 37.9
HDL, mg/dL	54.1 ± 6.5
AST, U/L	26.2 ± 12.6
ALT, U/L	24.4 ± 21.5
LDH, mg/dL	383.6 ± 146.3
24-h urine creatinine, g/mmol	917.2 ± 238.3
24-h urine protein, g/mmol	723.8 ± 356.5
24-h urine volume, g/mmol	1745.8 ± 501.9
Intima–media thickness of the right carotid artery, mm	0.55 ± 0.09
Intima–media thickness of the left carotid artery, mm	0.54 ± 0.10

BMI: body mass index; Hb: hemoglobin; PLT: platelets; ALT: alanine aminotranferease; AST: aspartate aminotransferase; ALP: alkaline phosphatase; CHOL: cholesterol; LDL: low-density lipoprotein; HDL: high-density lipoprotein, TG: triglyceride. LHD: Lactate dehydrogenase

**Table 2:** Basic characteristics of the population in terms of percentage and frequency.

Variable	Status	Frequency (%)
Gravid	One	11 (26.2)
	Two	13 (31)
	Three	10 (23.8)
	Four	5 (11.9)
	Five	3 (7.1)
Parity	Zero	15 (35.7)
	One	12 (28.6)
	Two	13 (31)
	Three	2 (4.8)
History of hypertension	Yes	4 (9.5)
	No	38 (90.5)
History of pre-eclampsia	Yes	7 (16.7)
	No	35 (83.3)
Non-alcoholic fatty liver	Yes	19 (45.2)
	No	23 (54.8)

**Table 3:** Basic characteristics of healthy pregnant women and pregnant women with pre-eclampsia in terms of mean and standard deviation.

Variable	Mean ± SD		Significance Level
	Healthy Pregnant Women	Pregnant Women with Pre-Eclampsia	
Mother’s age (years)	30.00 ± 5.92	32.86 ± 4.95	0.098
Gestational age (weeks)	33.57 ± 3.22	33.76 ± 2.57	0.833
BMI (kilograms per square meter)	30.65 ± 3.41	32.30 ± 3.75	0.144
Systolic blood pressure (mmHg)	102.86 ± 8.30	125.71 ± 13.53	>0.001
Diastolic blood pressure (mmHg)	64.76 ± 7.50	83.81±13.59	>0.001
Hb	12.53 ± 1.14	12.33 ± 1.14	0.624
PLT	18,361.05 ± 4187.80	202,142.8 ± 665,051.74	0.279
TG	322.14 ± 91.15	316.62 ± 125.49	0.871
CHOL	191.24 ± 36.69	243.67 ± 64.91	0.003
LDL	121.24 ± 21.72	134.33 ± 48.6	0.267
HDL	54.38 ± 7.83	54.24 ± 5.90	0.947
AST	20.76 ± 5.00	31.48 ± 15.60	0.005
ALT	13.90 ± 7.08	29.19 ± 32.59	0.042
LDH	370.49 ± 158.92	397.42 ± 136.00	0.558
24-h urine protein	85.59 ± 113.19	599.90 ± 972.2	0.027
24-h urine creatinine	908.86 ± 175.50	925.38 ± 292.73	0.826
24-h urine volume	1942.65 ± 396.99	1548.90 ± 526.88	0.009
Intima–media thickness of the right carotid artery	0.51 ± 0.08	0.60 ± 0.07	0.001
Intima–media thickness of the left carotid artery	0.10 ± 0.50	0.59 ± 0.09	0.003

**Table 4:** Basic characteristics of pregnant women and pregnant women with pre-eclampsia based on the frequency.

Variable	Frequency (%)			Significance Level
	Status	Healthy Pregnant Women	Pregnant Women with Pre-eclampsia	
Gravid	One	5 (23.8)	28.6 (6)	0.859
	Two	8 (38.1)	23.8 (5)	
	Three	5 (23.8)	23.8 (5)	
	Four	2 (9.5)	14.3 (3)	
	Five	1 (4.8)	9.5 (2)	
Parity	Zero	8 (38.1)	33.3 (7)	0.479
	One	7 (33.3)	23.8 (5)	
	Two	6 (28.6)	33.3 (7)	
	Three	0.0 (0)	9.5 (2)	
History of hypertension	Yes	0.0 (0)	19.0 (4)	0.053
	No	100 (21)	81.0 (17)	
History of pre-eclampsia	Yes	0.0 (0)	33.3 (7)	0.004
	No	100 (21)	66.7 (14)	
Non-alcoholic fatty liver	Yes	23.8 (5)	66.7 (14)	0.006

**Table 5:** Linear regression results regarding the right carotid artery intima–media thickness.

Variable	Beta Coefficient	Student’s *t*	Significance Level
Mother’s age (years)	0.146	0.922	0.328
BMI (kilograms per square meter)	0.023	0.171	0.865
CHOL	0.172	0.978	0.335
AST	−0.383	−1.240	0.223
ALT	0.340	1.113	0.273
Group (healthy or with pre-eclampsia)	0.434	2.582	0.014

**Table 6:** Linear regression results regarding the left carotid artery intima–media thickness.

Variable	Beta Coefficient	Student’s *t*	Significance Level
Mother’s age (years)	−0.064	−0.410	0.685
BMI (kilograms per square meter)	0.008	0.054	0.957
CHOL	−0.121	−0.649	0.521
AST	−0.105	−0.322	0.750
ALT	0.372	1.145	0.260
Group (healthy or with pre-eclampsia)	0.439	2.463	0.019

**Table 7:** Logistic regression findings with respect to the outcomes of non-alcoholic fatty liver.

Variable	Test Wald	The Significance Level	Odds Ratio (Confidence Interval)
Mother’s age (years)	1.933	0.164	1.107 (1.278-0.959)
BMI (kilograms per square meter)	0.456	0.500	1.076 (1.330-0.870)
History of preeclampsia	0.512	0.474	0.462 (3.824-0.056)
CHOL	1.337	0.248	1.010 (1.028-0.993)
AST	0.441	0.507	1.048 (1.205-0.912)
ALT	1.440	0.230	0.959 (1.027-0.895)
Group (healthy or with pre-eclampsia)	2.677	0.102	4.754 (30.771-0.735)
